# The Effect of Immunosuppressive Drugs on MDSCs in Transplantation

**DOI:** 10.1155/2018/5414808

**Published:** 2018-07-03

**Authors:** Fan Yang, Yang Li, Qian Zhang, Liang Tan, Longkai Peng, Yong Zhao

**Affiliations:** ^1^State Key Laboratory of Membrane Biology, Institute of Zoology, Chinese Academy of Sciences, Beijing, China; ^2^University of Chinese Academy of Sciences, Beijing, China; ^3^Center of Organ Transplantation, Second Xiangya Hospital of Central South University, Changsha, China

## Abstract

Myeloid-derived suppressor cells (MDSCs) are a group of innate immune cells that regulates both innate and adaptive immune responses. In recent years, MDSCs were shown to play an important negative regulatory role in transplant immunology even upstream of regulatory T cells. In certain cases, MDSCs are closely involved in transplantation immune tolerance induction and maintenance. It is known that some immunosuppressant drugs negatively regulate MDSCs but others have positive effects on MDSCs in different transplant cases. We herein summarized our recent insights into the regulatory roles of MDSCs in transplantation specially focusing on the effects of immunosuppressive drugs on MDSCs and their mechanisms of action. Studies on the effects of immunosuppressive drugs on MDSCs will significantly expand our understanding of immunosuppressive drugs on immune regulatory cells in transplantation and offer new insights into transplant tolerance. We hope to emphasize our concern for the negative effects of immunosuppressive agents on MDSCs, which may potentially attenuate the immune tolerance induction in transplanted recipients.

## 1. Introduction

Since the introduction of powerful immunosuppressive drugs like calcineurin inhibitors into treatment of allograft rejection, excellent short-term graft survival has been achieved. But chronic rejection and side effects of immunosuppressant like infection, malignancy, and drug toxicity still need to be solved urgently [1]. Cellular immunotherapy has made great achievements in cancer recently [2]. So, there is increasing interest in the potential of immune regulatory cells as cell therapy for transplant rejection. This is also a very promising solution for getting the “holy grail” of transplantation. Myeloid-derived suppressor cells (MDSCs) are immune regulatory cells studied most extensively in cancer; now, we know that MDSCs can also exert immune modulatory effects in transplantation [3]. In this review, we will discuss how these cells are induced and activated during different types of transplantation and the mechanism they employed to protect the graft or induce tolerance. Recently, there are already some attempts to induce MDSCs in vitro for administration to organ transplant recipients to promote graft survival and induce immune tolerance in animal transplant models. Nowadays, there are no clinical trials for MDSC-based cell therapy in transplantation. It is promising to further improve MDSC-inducing strategy with enhanced function for their clinical application. It will also be helpful for us if these cells can be manipulated in vivo to exert stronger and more specific suppressive function. Targeting MDSCs in transplant recipients for long-term survival even tolerance is promising but also challenging. Understanding how currently used immunosuppressive drugs acting on MDSCs will give lots of benefits for the future clinical medication, which may reduce side effects of high doses of immunosuppressive drugs and promote graft survival in transplantation. More importantly, it may shed lights on new treatment strategy targeting MDSCs to enhance their alloimmune suppressive capacity and promote tolerance in transplantation.

## 2. MDSCs

MDSCs are a class of immune-negative regulatory cells, with the earliest found in the late 70s of the last century. BCG inoculation or systemic irradiation of mice can result in inflammatory response, which will induce a group of abnormally proliferating myeloid cells with the ability to inhibit the activation and function of cytotoxic T cells, known as natural suppressor cells [4]. In recent years, MDSCs have been reported mainly in a variety of tumor animal models and patients, and the concept introduction of MDSC is mainly to describe these myeloid cells under the conditions of abnormal activation. But many other inflammatory microenvironments, such as trauma, chronic infections, acute infection-induced sepsis, tissue damage caused by radiation, and autoimmune diseases, also have similar cells. Under different activation conditions, MDSCs mediate immune-negative regulation through different mechanisms [5]. MDSCs are essentially a heterogeneous population of early myeloid progenitors, immature granulocytes, macrophages, and dendritic cells (DCs) at different stages of differentiation. Usually, MDSCs were divided into two subgroups: monocytic (M-MDSCs) and granulocytic (G-MDSCs) [6]. Since G-MDSCs actually had less granules and low density, also with a distinctive phenotype from neutrophils, it is recommended to be named as polymorphonuclear- (PMN-) MDSCs recently as the standard nomenclature [7]. These two subsets can be distinguished by surface markers [8]. In mice, M-MDSCs were characterized by phenotypic markers as CD11b^+^Ly6G^−^Ly6C^high^ and PMN-MDSCs as CD11b^+^Ly6G^+^Ly6C^low^. In humans, M-MDSCs were defined as CD11b^+^CD14^+^CD15^−^HLA-DR^−/low^ and PMN-MDSCs as CD11b^+^CD14^−^CD15^+^ (or CD66b^+^). Both of them were from human peripheral blood mononuclear cells (PBMCs) to exclude mature neutrophils. In human PBMCs, lin^−^ (including CD3, CD14, CD15, CD19, and CD56) HLA-DR^−^CD33^+^ cells containing mixed groups of MDSCs with more immature progenitors have been defined as early-stage MDSCs (e-MDSCs), which have no equivalent in mice [7]. It should be pointed out that we do not have specific markers to define MDSCs and their subpopulations so far. This important issue requires to be addressed in the future.

Two major groups of MDSCs often use different mechanisms to mediate immunosuppression in tumor models, with high levels of nitric oxide synthase 2 (NOS2 or iNOS) for M-MDSCs and reactive oxygen species (ROS) for PMN-MDSCs. But both of them can rely on arginase 1 (Arg1) for suppression. Arg1 and iNOS deplete L-arginine from microenvironments, and either together, or separately, they subsequently block the translation of the T cell CD3*ζ* chain, inhibit T cell proliferation, and promote T cell apoptosis [9]. ROS produced by PMN-MDSCs reacts with NO to induce nitration and nitrosylation of amino acid in molecules of T cell signaling for functional inhibition [10]. Other mechanisms are also involved in immunosuppression in addition to the ones mentioned above. Indoleamine 2,3-dioxygenase (IDO) is an important immune regulatory enzyme for environmental tryptophan consumption to induce T cell dysfunction, which has been well documented in DCs and macrophages [11, 12]. MDSCs can also induce IDO expression in cancer [13]. LPS-induced MDSCs suppress immune response by heme oxygenase-1 (HO-1) through IL-10 [14]. TGF-*β* and IL-10 produced by MDSCs can mediate the cytotoxic NK cell inhibition and Treg cell induction. ADAM metallopeptidase domain 17 on MDSCs can cut CD62L and thus inhibit the recognition of T cells with antigen-presenting cells (APCs) in lymph nodes [15]. Galectin 9 (GAL9) on MDSCs can act directly on T cell immunoglobulin- and mucin-domain-containing molecule-3 (TIM3) on T cells to mediate their apoptosis [16]. Upregulating prostaglandin E2 (PGE2) by cyclooxygenase-2 (COX2) expression was also employed by MDSCs for immune suppression in tumor condition [17].

**Figure 1:**
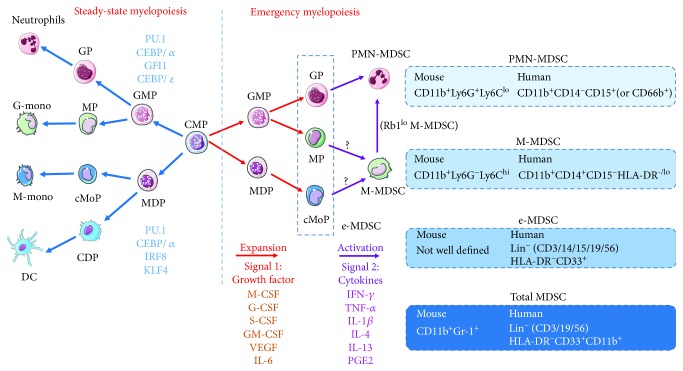
The development, subsets, and phenotypes of MDSCs. MDSCs arise from CMP in the presence of several growth factors and cytokines during emergency myelopoiesis under inflammatory conditions. Growth factors (signal 1) drive the expansion of myeloid cell progenitors. Subsequent activation signal (signal 2) via cytokines endows these progenitors with immunosuppressive function to give rise to e-MDSCs, M-MDSCs, and PMN-MDSCs. Recently, it was found that GMP and MDP yielded distinct monocyte-committed progenitors which differentiated into different monocyte subsets at steady-state, respectively. Which of the two monocyte-committed progenitors can give rise to functional M-MDSCs and further acquiring the ability to differentiate into PMN-MDSCs during emergency myelopoiesis is unclear. The phenotype markers of different MDSC subsets are illustrated here. CMP, common myeloid progenitor; GMP, granulocyte-monocyte progenitor; MDP, monocyte-dendritic cell progenitor; MP, monocyte-committed progenitor; cMoP, common monocyte progenitor; GP, granulocyte-committed progenitor; G-mono, GMP-derived monocyte; M-mono, MDP-derived monocyte.

The development and accumulation of MDSCs are mainly dependent on two types of signals. The first signal is responsible for immature myeloid cell expansion, and the second is for their pathologic activation in emergency myelopoiesis [18]. MDSCs arise from lineage-committed progenitors including common myeloid progenitors (CMPs) and granulocyte-monocyte progenitor (GMP) downstream of hematopoietic stem and progenitor cells [19]. Recently, it was found that monocyte-dendritic cell progenitors (MDPs) arose from CMPs independently of GMPs, GMP-, and MDP- produced monocytes via similar but distinct monocyte-committed progenitors [20]. It is interesting to clarify the developed pathway of M-MDSCs from each progenitor. Studies also showed that epigenetic silencing of the retinoblastoma (Rb) gene controlled by histone deacetylase 2 (HDAC-2) promotes monocyte preferential differentiation towards PMN-MDSCs (Figure 1) [21, 22]. Growth factors like GM-CSF, G-CSF, and M-CSF work as expansion signals, and cytokines like IFN-*γ*, IL-1*β*, IL-6, IL-10, IL-12, and IL-13 are responsible for their pathologic activation. The first group of signals activated downstream transcription factors or pathways like STAT3, IRF8, C/EBP*β*, RB1, Notch, adenosine receptors A2b signaling, and NLRP3 for MDSC expansion [23]. The second group of signals employs NF-*κ*B pathway, STAT1, STAT6, PGE2, and COX2 for full function [24].

## 3. MDSCs in Transplantation and Mechanisms of Immune Suppression

### 3.1. MDSCs in Organ Transplantation

The concept of MDSCs was introduced into transplantation field to describe similar phenotypic cells found in cancers where they were intensively studied. Under tumor conditions, MDSCs can be divided into M-MDSCs and PMN-MDSCs; these two groups of MDSCs have some differences in development, phenotype, and mechanisms mediating immune suppression [6]. This is also true in different transplant models (Table 1). In most cases, M-MDSCs play more important roles in transplant tolerance induction and graft protection. For example, CD11b^+^CD115^+^Gr1^+^ M-MDSCs promote tolerance by iNOS through IFN-*γ* and STAT-1 in a mouse heart transplant model [25]. CD11b^+^CD33^+^HLA-DR^−^CD14^+^ M-MDSCs from kidney-transplanted patients can inhibit CD4^+^ T cell proliferation and expand Treg cells in mixed leukocyte reactions in vitro [26].

Clinical significance of MDSCs in human renal transplantation with acute T cell-mediated rejection was confirmed by comparing patients with higher MDSCs in PBMCs to the lower ones. Patients with high levels of MDSCs had better graft function and longer organ survival time [27]. In intestinal transplantation, MDSCs accumulate in the recipient PBMCs and the grafted intestinal mucosa, and MDSC numbers decreased in intestinal transplant recipients suffering from acute cellular rejection, which suggests that MDSC may regulate acute cellular rejection [28].

In animal transplant models, whether or not MDSCs can be induced in the absence of any immunosuppressive treatment is controversial. In our group, we found that MDSCs with suppressive capacity can be induced in mouse spleen by alloskin transplantation [29]. But data from other groups using mouse heart transplantation model supports that functional MDSCs cannot be induced by transplantation alone [25, 30]. This may be due to the different intensity of the alloimmune response in different models. The earliest report on the role of MDSCs in organ transplantation is in the rat kidney transplant model treated with anti-CD28 mAb. MDSCs expressing CD11b and Sirp*α* in blood and bone marrow inhibit T cell proliferation, but the counterparts in lymph nodes and spleen cannot [31]. The mechanism of these MDSC-mediated inhibitions is through the iNOS, since the addition of iNOS inhibitor rescues T cell proliferation in vitro and restores the survival time of the graft in vivo [31]. Subsequent study from the same group demonstrated that expression of CCL5 by MDSCs in the blood was downregulated, while the expression level in the graft was unchanged, which promoted the recruitment of Treg cells into the graft and supported graft survival [32]. In a mouse heart transplant model, donor-specific transfusion (DST) + anti-CD40L treatment induced accumulation of CD11b^+^Gr-1^+^CD115^+^ MDSCs in blood and bone marrow and CD11b^+^Gr-1^+^ MDSCs in allografts. But only MDSCs in allograft can suppress T cell proliferation in MLR. Using *Ccr2*^−/−^ mice which cannot induce tolerance by DST + anti-CD40L, it was found that a transfer of CD115^+^CD11b^+^Gr1^+^ bone marrow monocytes can restore tolerance but not monocytes from *Ifngr*^−/−^, *Nos2*^−/−^, *Stat1*^−/−^, or *Irf-1*^−/−^ mice. This demonstrated that IFN-*γ* to iNOS signaling pathway was necessary for MDSC function [25].

Immunoglobulin-like transcript 2 (ILT2) is an inhibitory receptor that is widely expressed on white blood cells. In ILT2 transgenic mice (ILT2 constitutively activated by mouse H2-D^b^), the ratio of CD11b^+^Gr-1^+^ MDSCs increased in both spleens and peripheral blood. Wild-type B6 mice and ILT2 transgenic mice were transplanted with bm2 mouse skin which had only one mismatch locus in MHC class II molecules. Six days later, MDSCs in spleens were sorted and transferred to B6 recipients transplanted with bm2 skin. MDSCs from ILT2 mice could promote graft survival significantly [33]. Our laboratory reported that Smad3-deficient mice were defective for skin and cardiac graft rejection with reduced T cell infiltration in the graft comparing to WT mice, but the numbers and function of MDSCs were upregulated. Functional enhancement for MDSCs in Smad3-deficient mice mainly relies on iNOS. MDSC depletion antibody RB6-8C5 reversed the protective effect on the graft survival for Smad3-deficient mice [34]. LPS tolerance-induced MDSCs have the ability to inhibit T cell proliferation in vitro. After transfer to recipient B6 mice grafted with bm12 skin, MDSCs prolong the graft survival through HO-1-dependent IL-10 production [14]. Peritonitis induced by cecal ligation and puncture results in MDSC accumulation in the bone marrow. After transfer to recipient mice, these cells reduced corneal neovascularization and promote graft survival in allocorneal transplantation model [35]. IL-33 treatment can prolong graft survival with increased CD11b^+^Gr-1^int^ MDSCs in allografts, spleens, and bone marrow in the bm12 to B6 heart transplant model. But whether IL-33 can directly induces MDSC expansion or activation needs to be illustrated [36]. Donor IL-6 deficiency also significantly prolongs graft survival with increased CD11b^+^Gr-1^low^ splenic MDSCs and graft infiltration of CD11b^+^Gr-1^low/int^ MDSCs in the B6 to BALB/c heart transplant model [37, 38]. G-CSF treatment in BALB/c mice can induce functional MDSCs in spleens which can suppress T cell proliferation in vitro. G-CSF can also prolong allograft survival in a bm12 to B6 skin transplant model with increased CD11b^+^Gr-1^+^ MDSCs in blood and spleen [39]. Hepatic stellate cells cotransplanted with alloislets can prolong graft survival with increased CD11b^+^CD11c^−^ MDSCs in spleen [40].

### 3.2. MDSCs in Transplant Tolerance

There are many ways to induce transplant tolerance in rodent animal models like costimulatory blockades or donor-specific transfusion [41]. Although it is difficult to repeat in large animals probably because of the presence of more memory T cells, these results are important for understanding the mechanisms of tolerance induction. MDSCs may be a key factor for transplant tolerance maintenance. In a renal transplant model, anti-CD28 treatment-induced tolerance can be interrupted by iNOS inhibitor, which suggests the role of MDSCs in tolerance maintenance [31]. Studies using a heart transplant model support this idea. In this model, graft tolerance was induced by anti-CD154 and DST treatment. Different types of myeloid cells were depleted during the transplantation by using anti-Gr-1, anti-Ly6G antibody, CD11b-DTR, and MaFIA mice, and the results showed that CD115^+^CD11b^+^Gr-1^+^ MDSCs recruited to heart grafts from bone marrow played a key role on tolerance maintenance [25]. Tolerance was independent of splenic MDSCs because mice with splenectomy can also induce tolerance. Treg cell induction and maintenance were dependent on MDSCs by monocyte depletion in vivo [25]. Further study showed that DC-SIGN signaling on M-MDSC-derived macrophages was required for tolerance induction in mouse heart transplantation by costimulatory blockade for tolerance induction [42]. Using anti-CD154 mAb and DST treatment for heart transplant tolerance induction, another study showed that *Listeria monocytogenes* infection can break the tolerance and cause acute graft rejection [43]. But the donor-specific tolerant state reemerges, allowing spontaneous acceptance of a donor-matched heart after the secondary transplantation [43]. As MDSCs play important roles for tolerance in this model, infection may disturb the function of MDSCs and lead to rejection. Spontaneous tolerance by secondary transplantation may also depend on the recovery of MDSC function. Transfusion of donor splenocytes treated with 1-ethyl-3-(3′-dimethylaminopropyl)-carbodiimide (ECDI-SPs) provides donor-specific tolerance of islet allografts. ECDI-SPs also significantly prolong cardiac allograft survival, and depletion of MDSCs or inhibition of IDO reversed this effect [44]. ECDI-SPs treatment increased both M-MDSCs and PMN-MDSCs in the spleen of allograft-transplanted recipients. Both of them can suppress T cell proliferation in vitro, and their protective effect for allograft was mediated in part by intrinsic IFN-*γ*-dependent mechanisms [45].

### 3.3. MDSCs in Hematopoietic Stem Cell Transplantation

Allogeneic hematopoietic stem cell transplantation (allo-HSCT) is an important therapeutic procedure to treat hematologic malignancies, which can cause graft-versus-host disease (GVHD). It is reported that circulating CD14^+^HLA-DR^−/low^ M-MDSCs with suppressive function mediated by IDO were increased in patients after allo-HSCT with GVHD [46]. Further study showed that the M-MDSCs in GVHD patient expressed CD1d and CD226, and CD1d^+^ M-MDSC exerted strong immune-suppressive effect [47]. MDSC subsets were recovered between 2 and 4 weeks after allo-HSCT; they can suppress T cell proliferation and promote Treg cell development [48]. In human haploidentical-HSCT, G-CSF plays an important role in MDSC induction. M/PMN/e-MDSCs expanded in bone marrow and peripheral blood of donors after G-CSF treatment, and M/e-MDSCs are important factors associated with the low risk of acute GVHD [49]. Early studies in mouse GVHD models support this idea. In a murine model of allogeneic bone marrow transplantation (BMT), GVHD was induced by donor lymphocyte infusions immediately after BMT. MDSCs expanded in this model with the ability to suppress alloreactive T cell proliferation in MLR via iNOS [50]. It was reported that CpG + IFA treatment of donor mice can induce the accumulation of MDSCs in peripheral blood and spleens, which then protected mice from GVHD [51]. MDSCs isolated from G-CSF subcutaneously injected mice can inhibit acute GVHD through an IDO-independent mechanism [52]. G-CSF treatment also generates a population of suppressive neutrophils with less granule content and low density (features of PMN-MDSCs), which reduce acute GVHD in an alloantigen-specific manner through IL-10 and Treg generation [53]. Mice with SHIP deficiency accept allo-HSCT without serious GVHD, which was due to the accumulation of MDSCs impairing alloreactive T cell priming [54]. Transplantation of bone marrow cells from MyD88-deficient C57BL/6 (B6) mice together with B6 T cells into MHC-matched allogeneic BALB.B strain mice can induce more serious GVHD than transfer with WT bone marrow cells. The aggravation of GVHD was associated with impaired expansion of CD11b^+^Gr1^+^ MDSCs from the MyD88-deficient bone marrow cells during the GVHD development [55]. The in vitro induced MDSCs by G-CSF + GM-CSF + IL-13 from bone marrow cells inhibit lethality caused by GVHD through Arg1 [56]. GM-CSF + G-CSF-induced MDSCs attenuate GVHD by skewing T cells toward type 2 T cells [57]. The function of these induced MDSCs can be further improved by inhibiting their inflammasome activation to inhibit GVHD lethality [58]. To investigate the regulatory role of myeloid cells in GVHD, subclinical GVHD model was constructed in nonirradiated F1 hybrids by transfer of parental splenocytes [59]. Both M-MDSC and PMN-MDSC subsets suppressed alloreactive T cell proliferation in vitro and in vivo [60]. These results collectively suggested that MDSCs may play immune regulatory roles in allo-HSCT to suppress GVHD.

### 3.4. Induction of MDSCs In Vitro

MDSCs can be induced in vitro for potential clinical application such as in organ transplantation. Early study reported that GM-CSF + LPS + IFN-*γ* can induce functional MDSCs in vitro and in vivo [61]. It is reported that the combination of GM-CSF and IL-6 was sufficient to induce MDSCs which prolonged alloislet graft survival after transfer to recipient mice [62]. These two cytokines can also induce functional MDSCs from human PBMCs [63]. This combination was further confirmed in a skin transplantation model. Repeated injection of MDSCs or a single injection of activated MDSCs by LPS stimulation resulted in prolonged allograft survival by short-term T cell exhaustion [64]. GM-CSF + IL-4 + PGE_2_ induce the differentiation of MDSCs with enhanced function from human monocyte isolated from human PBMCs [65]. MDSCs induced by GM-CSF alone or M-MDSCs induced by M-CSF + TNF*α* can also prolong the survival of skin grafts with HY antigen [29, 66]. Functional MDSCs induced by GM-CSF + IL-4 prolonged alloislet survival after cotransplantation via iNOS [67, 68]. B7-H1 was required for MDSCs to exert immune regulatory activity and induction of Treg cells in this model [69].

## 4. Clinically Used Immunosuppressive Drugs and Their Effects on MDSCs

There are five major categories of clinical immunosuppressive agents (Table 2). Herein, we briefly discuss their mechanism of action to mediate immunosuppression and their effects on MDSCs.

### 4.1. Corticosteroids (CS)

CS including glucocorticoids (GC) and mineralocorticoids (MC) are the product of adrenal cortex, with broad-spectrum immunosuppressive and anti-inflammatory effects. Clinically applied CS are mainly GC which can activate gluconeogenesis. GC can enter the cell membrane in two ways. Unbound GC can passively diffuse into cell membrane, and they can also enter the cell via membrane receptor after binding with corticosteroid-binding globulin (CBG) [70]. GC can bind to glucocorticoid receptors (GR) then promote many gene activation by binding to glucocorticoid response element DNA sequence. Different chromatin accessibility determined that GR regulated different genes in different cell types [71]. GR regulate the immune response by interacting with other transcription factors without its own direct DNA binding. Many proinflammatory transcription factors like nuclear factor­*κ*B (NF­*κ*B), activator protein 1 (AP­1), the signal transducer and activator of transcription (STAT), CCAAT/enhancer­binding protein (C/EBP), and nuclear factor of activated T cells (NFAT) can interact with GR [72]. GC inhibit the initiation of inflammation and cell recruitment to inflammatory sites and promote the resolution of inflammation [73]. At the initial stage of the inflammation, GC can inhibit downsteam signaling of pattern recognition receptors. For example, GC can upregulate dual­specificity protein phosphatase 1 (DUSP1) to inhibit mitogen­activated protein kinase 1 (MAPK1) and IL­1 receptor­associated kinase 3 (IRAK3) signaling downstream of TLR and IL-1 receptor signaling [74]. GC inhibit eicosanoid production by macrophages to reduce vascular permeability [75]. Ligated GR can bind to mRNA of CCL2 and CCL7 to promote their degradation [76]. GC promote phagocytosis of macrophages and monocytes for apoptotic cells and debris to accelerate the resolution of inflammation [77]. For adaptive immunity, GC influence T cell activation by inhibiting DC maturation and upregulating IL-10 expression [78]. GC directly inhibit TCR signaling by disturbing the activity of AP­1, NF­*κ*B, and NFAT [79]. But GC increase peripheral Treg cell frequency by targeting glucocorticoid-induced leucine zipper (GILZ) [80, 81].

**Figure 2:**
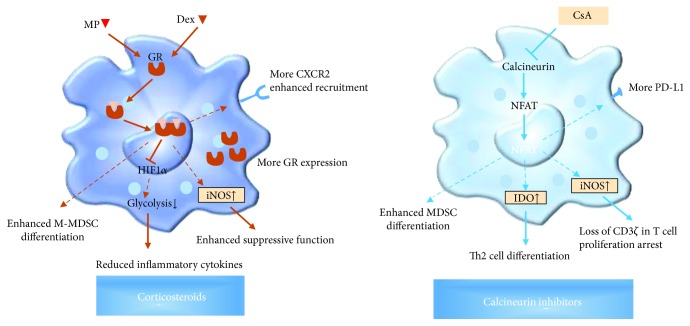
The modulatory effects of corticosteroids and calcineurin inhibitors on MDSCs. The effects of corticosteroids and calcineurin inhibitors on MDSCs were illustrated here. Targeting GR and calcineurin by corticosteroids and CsA, respectively, altered MDSC differentiation, suppressive function, and recruitment. MP, methylprednisolone; Dex, dexamethasone; GR, glucocorticoid receptors; HIF1*α*, hypoxia-inducible factor 1 *α*; iNOS, inducible nitric oxide synthase; IDO, indoleamine 2,3-dioxygenase; CsA, cyclosporin A.

CS are important immunosuppressive drugs for organ transplant medication at early times, which are now often used in early induction therapy stages. Prednisone and methyl-prednisolone were CS commonly used in clinics, and they were also the earliest drug used to inhibit transplant rejection. CS can directly target monocytes/macrophages to inhibit IL-12 production, which subsequently redirects T cell polarization from Th1 to Th2 cells [82, 83]. CS also strongly inhibit the production of IL-12p70, TNF-*α*, and IL-6 by LPS-stimulated monocyte-derived immature DCs (iDCs) in vitro [84]. GC did not cause a global effector function suppression of monocyte but result in differentiation of monocytes with a specific anti-inflammatory phenotype [77]. Dexamethasone- (Dex-) treated monocytes can upregulate CD163 and Gr-1 with a phenotype like M-MDSCs [85]. Dex profoundly modulates CD40-dependent DC activation by downregulating costimulatory, adhesion, and MHC class I and II molecules and without expressing the maturation marker CD83. Dex also suppressed the production of the proinflammatory cytokine IL-12 and potentiated the secretion of the anti-inflammatory cytokine IL-10 without affecting antigen uptake [86]. Dex inhibits the development and maturation of BMDCs from human monocytes treated with GM-CSF and IL-4 for 7 days [87]. In a mouse immunological hepatic injury model by LPS shock, MDSCs display significantly lower levels of GR. Dex treatment can restore GR expression in MDSCs and enhance the suppressive function by suppressing HIF1*α* and glycolysis [88]. In a mouse skin transplant model, Dex can relieve graft rejection. By upregulating the expression of CXCL1 and CXCL2 chemokines in the graft, more CD11b^+^Gr-1^+^ MDSCs were recruited into skin grafts. Removal of these cells with anti-Gr-1 depletion antibodies or glucocorticoid receptor antagonist treatment reversed the mitigation effect of Dex on skin graft rejection, indicating that binding of Dex directly to glucocorticoid receptor mediated the accumulation and inhibitory function of MDSCs to promote graft survival. Dex-treated MDSCs promote Th2 cell differentiation to alleviate graft rejection through iNOS [89]. MDSCs from Dex-treated mice transferred to unmanipulated recipients can prolong alloskin graft survival, but MDSCs from untreated alloskin-grafted mice cannot. Thus, Dex can initiate the accumulation of MDSCs in spleens of alloskin-grafted mice and endow these cells with the immunosuppressive function. In another study, it was found that Dex treatment on GM-CSF-induced MDSCs in vitro increase the number of CD11b^+^Gr-1^int/low^ MDSCs with an enhanced immunosuppressive function. Adoptive transfer of these MDSCs significantly prolonged heart allograft survival and also favored the expansion of Treg cells in vivo. Mechanistic studies showed that iNOS signaling was required for suppressive function of MDSCs. GR signaling played a critical role in the recruitment of transferred MDSCs into allografts through upregulating CXCR2 expression on MDSCs [90]. In PBMCs of intestinal transplant recipients, MDSC numbers were positively correlated with serum IL-6 levels and the glucocorticoid administration index. IL-6 and methylprednisolone treatment enhanced the differentiation of bone marrow cells to MDSCs in vitro [28]. Therefore, CS exert positive modulatory effects on MDSCs in transplanted recipients (Figure 2).

### 4.2. Calcineurin Inhibitors (CNIs)

CNIs include a class of drugs targeting at calcineurin, and the most commonly used ones are cyclosporin A (CsA) and tacrolimus (FK506). CNIs become the mainstream medication for organ transplantation since the introduction of CsA to this field [91]. CsA and FK506 bind to different immunophilins as cyclophilins and FK-binding proteins, respectively. Then the complex binds to an intracellular molecule calcineurin, which is a protein phosphatase for cytoplasmic NFAT dephosphorylation and its subsequent translocation to nucleus to perform function. NFAT is a key transcription factor by upregulating many cytokines and costimulatory molecules, like IL-2, IL-4, TNF-*α*, and CD40 ligand, for full activation of T cells [92]. However, CNIs also have a negative effect on the proliferation and function of Treg cells due to impaired function of NFAT [93, 94].CNIs also regulate innate immune cells. CsA inhibits the activation of neutrophils stimulated by angiotensin II through the MAPK and ERK pathways [95]. Calcineurin inhibition by FK506 leads to decreased responsiveness to LPS in macrophages and dendritic cells [96]. CNIs inhibit expression of iNOS in macrophage cell lines [97]. CNIs also have effect on parenchymal cells, and it is well known that CNIs have toxicity to endothelial cells [98].

Because targets of CNIs are NFAT and MAPK pathways, which are widely used signaling by myeloid cells, it is not surprising that CNIs affect myeloid cell functions including MDSCs. Tacrolimus impairs clearance of fungal pathogen *Aspergillus fumigatus* from the airway by targeting TLR9-BTK-calcineurin-NFAT pathway in macrophage [99]. Treatment of bone marrow-derived macrophages (BMDMs) with tacrolimus significantly inhibited LPS and LPS plus IFN-*γ*-induced IL-12p40 mRNA and protein expression [100]. After coculture with increasing concentrations of CsA for 24 h, the number of live splenic MDSCs decreased significantly in a dose-dependent manner by calcineurin inhibition [101]. In the mouse skin transplant model, a daily dose of 15–30 mg/kg of CsA can promote the accumulation of CD11b^+^Gr-1^+^ MDSCs in the graft, draining lymph nodes, spleen, peripheral blood, and bone marrow with the prolonged survival time of grafts [102]. The expression of CXCR2 was upregulated on splenic MDSCs [102]. Blocking this receptor or removal of these cells by anti-Gr-1 depletion antibody reverses the mitigation effect of CsA on transplant rejection [102]. Adoptive transfer of MDSCs from spleens of CsA-treated skin-grafted mice to newly transplanted mice promotes graft survival [102]. CsA promotes the immunosuppressive function by downregulating NFATc1 in MDSCs, thereby promoting the differentiation of Th cells into Th2 cells. MDSC depletion reverses the tendency of T cell polarization [102]. Finally, the authors demonstrated that CsA regulated MDSC function via calcineurin-NFAT-IDO axis [102]. In our group, the effects of CsA on MDSC differentiation and development were explored in vitro and in vivo [103]. CsA treatment significantly increases the number, phenotype, and function of GM-CSF-induced MDSCs by in vitro assays. Similar results were obtained in alloskin-grafted mice with CsA administration. The enhanced immunosuppressive function of MDSCs is related to the upregulation of iNOS and CD274 [103]. Thus, CNIs may regulate MDSC differentiation and immunosuppressive function by NFAT (Figure 2).

### 4.3. mTOR Inhibitors (mTORi)

mTORi is targeting at the protein named mTOR (mechanistic target of rapamycin). mTOR is a serine, threonine protein kinase, which is the main component of two complexes that mediate different signal transduction named mTORC1 and mTORC2. Rapamycin can bind to FK506-binding protein 12 (FKBP12) to form an immunosuppressive complex to inhibit mTOR. mTORC1 plays a central role in regulating cell processes for anabolism in response to environmental conditions. mTORC1 promotes protein synthesis largely through the phosphorylation of p70S6 kinase 1 (S6K1) and eIF4E-binding protein (4EBP). mTORC1 promotes lipid and nucleotide synthesis by different mechanism. mTORC1 provides substrates for anabolism by promoting glycolysis. mTORC2 controls cell proliferation and survival by downstream effector molecules like protein kinase PKA/PKG/PKC to regulate cytoskeletal remodeling. Akt can be phosphorylated and activated by mTORC2 to promote cell survival and proliferation through FoxO1/3a, GSK3*β*, and TSC2 downstream of Akt. mTORC2 also phosphorylates and activates SGK1 to control ion transport and cell survival. The role of mTOR in innate and adaptive immunity has been well reviewed [104–106], so we will not discuss it excessively here.

**Figure 3:**
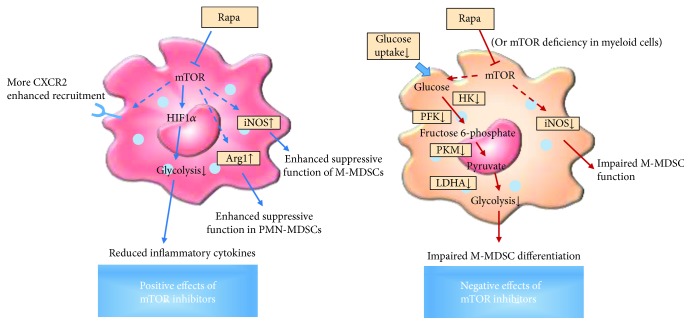
The different modulatory effects of mTOR inhibitors on MDSCs. The regulatory effects of mTOR inhibition on MDSCs were controversial so far. The positive and negative effects of rapamycin on MDSCs were both illustrated here. This inconsistency may be due to different animal models or different doses and modes of rapamycin administration. Rapa, rapamycin; Arg1, arginase 1; iNOS, inducible nitric oxide synthase; HK, hexokinase; PFK, phosphofructokinase; PKM, pyruvate kinase muscle isozyme; LDHA, lactate dehydrogenase-*α*.

The mTOR signaling significantly affects the development of myeloid cells. It masters monocyte/macrophage development at the early stages through regulating STAT5-IRF8-dependent CD115 expressing pathway [107, 108]. Inhibition of mTOR by rapamycin promotes inflammatory cytokine production through NF-*κ*B but blocks IL-10 via STAT3 on human monocyte [109]. mTOR inhibition by rapamycin interferes GC signaling and prevents the anti-inflammatory potency of GC in human monocytes [110]. We found that rapamycin treatment reduced cell number of M-MDSCs in a skin transplantation model [29]. The suppressive function of M-MDSCs from spleens of recipients in vitro was also impaired by rapamycin treatment [29]. Using myeloid-specific mTOR-deficient mice, we obtained similar results with rapamycin treatment [29]. Rapamycin treatment also undermines the differentiation, proliferation, and function of GM-CSF-induced MDSCs in vitro [29]. Finally, it was demonstrated that inhibition of glycolysis and subsequent downregulation of iNOS were the main mechanisms of rapamycin affecting MDSCs [29]. In murine immunological hepatic injury model by injection of ConA, inhibition of mTOR by rapamycin enhanced suppressive function of liver MDSCs and promoted MDSC recruitment to inflammatory site via iNOS [111]. Mechanism studies show that MDSCs suppress T cell activation and modulate T cell differentiation by targeting the HIF1*α*-dependent glycolytic pathway [112]. It is also reported that SIRT1 can regulate MDSC differentiation to M2 phenotype by blocking HIF1*α*-dependent glycolysis and rapamycin recovers MDSC suppressive function by blocking glycolytic activity in SIRT1 KO cells [113]. In the acute kidney injury mouse model, inhibition of mTOR signaling by rapamycin promotes MDSC recruitment and enhances PMN-MDSC development and suppressive function of MDSCs to ameliorate acute kidney injury [114]. In another study, rapamycin treatment in the mouse heart transplant model increased the number and function of MDSCs, and depletion of these cells by anti-Gr-1 antibody reversed the mitigation effect of rapamycin. M-MDSCs and PMN-MDSCs were isolated from the spleen of transplant recipients. Both subsets of MDSCs treated with rapamycin had the ability to inhibit T cell proliferation, and the immunosuppression was mediated by upregulation of iNOS and Arg1, respectively [30]. In untreated group, MDSCs have no suppressive function. PMN-MDSCs or M-MDSCs from rapamycin-treated mice were administered to newly heart-transplanted recipient via the inferior vena cava or the aorta of the transplanted heart, and both of them prolong graft survival and the effect of M-MDSCs was more pronounced. But MDSCs from PBS-treated mice have no effect [30]. Current reports on the roles of mTORi on MDSCs are not consistent. The reason for this inconsistency is whether caused by different transplant models or different treatment protocols which need further elucidation (Figure 3).

### 4.4. Purine Analogues

Purine analogues are compounds structurally similar to DNA and RNA synthetic substrates that can interfere with the synthesis of DNA, RNA, and other nucleic acids to inhibit cell proliferation and immune responses. Azathioprine (AZA) and 6-mercaptopurine (6-MP) are widely used immunosuppressive agents to prevent transplant rejection. Actually from the early 60s to the early 80s, AZA and steroids are the main medication for transplant rejection [115]. Mycophenolate mofetil (MMF) is another drug acting on purine synthesis pathway with mycophenolic acid as its active metabolite of MMF. Through inhibiting inosine monophosphate dehydrogenase (IMPDH), which mediated the only pathway for lymphocyte guanosine nucleotide synthesis, MMF can suppress lymphocyte proliferation specially. So MMF has substituted AZA for transplant medication in recent years [116].

There are no reports on the role of antiproliferative drugs on MDSCs so far. But this drug might potentially inhibit the development of MDSCs, because MPA has been reported to suppress granulopoiesis [117]. In kidney transplant recipients with long-term stable graft function, MMF treatment reduces the production of IL-1*β*, IL-10, and TNF-*α* by monocytes [118]. MMF also inhibits upregulation of ICAM-1 and MHC-II expression on human monocytes by LPS or IFN-*γ* stimulation and the adhesion of monocyte to endothelial cells [119]. Human monocyte-derived DCs can be induced by GM-CSF + IL-4 treatment in vitro. MMF can impair their differentiation, maturation, and allostimulatory function [120]. MPA inhibits IL-1*β* production by human CD14^+^ monocytes stimulated by PMA/ionomycin [121]. The effects of MMF on MDSCs should be addressed in the near future.

### 4.5. Costimulatory Blockade

Costimulatory blockade is a common method to induce tolerance in animal models of transplantation. Belatacept is the first and only currently used immunosuppressive drug for treatment of rejection in renal transplantation as a costimulatory blocker. CD28-mediated costimulatory signals are essential for the survival, proliferation, and cytokine production of T cells. B7-1 (CD80) and B7-2 (CD86) expressed on the surface of APCs are the main ligands of CD28. CTLA-4 is a negative regulatory molecule sharing the same ligands with CD28 on T cell surface. CTLA-4 expression is lagging behind CD28 but with more affinity than CD28 to B7. CTLA-4 has a stronger affinity for B7-1 which is also expressed at a later phase of T cell activation on APCs [122]. CTLA-4 and IgG Fc fragment were fused into CTLA-4Ig, which can block CD28 signaling with higher affinity for B7-1/2. Two amino acids were mutated in CTLA-4Ig for enhancing the binding ability to B7-2 which resulted in the generation of belatacept [123]. The 7-year-phase-III clinical trial found that the use of belatacept-based renal transplantation therapy was associated with lower nephrotoxicity compared to CNI-based group, and the proportion of patients who produced anti-HLA antibodies after transplantation was lower than CNI treatment group [124]. Recently, ASP2409, a next-generation of CTLA4-Ig with 14-fold higher binding affinity with CD86 than belatacept in vitro had exhibited potent suppressive effects on the monkey renal transplantation model without serious side effects [125]. FR104, an antagonist anti-CD28 monovalent Fab′ antibody, was proved to show preclinical efficacy and immunological safety in 2012 [126]. FR104 prevented acute rejection and alloantibody development with low doses of tacrolimus in the nonhuman primate renal transplantation in 2015 [127]. FR104 and belatacept exert different effects on mechanisms of renal allograft rejection in baboons [128]. Study in healthy human subjects with FR104 reported in 2016 and results showed that FR104 has potential to use in clinics for transplantation [129]. In mouse and nonhuman primate transplant models, blocking CD40/CD40L pathway is more effective for allograft survival and tolerance induction comparing to CD28 blockade [130]. Unfortunately, anti-CD40L in clinical trials showed a number of thromboembolic complications. Recently, it is reported that a novel anti-CD154 mAb that lacks Fc-binding activity was safe without evidence of thromboembolism. It is equally as potent as previous anti-CD154 agents at prolonging renal allograft survival in a nonhuman primate preclinical model [131]. Thus, it is promising that the costimulatory blockades will be widely used in clinics to avoid graft rejection and even immune tolerance induction in transplanted patients.

Costimulatory blockade can effectively inhibit T cell activation by blocking the secondary signals, which can promote T cell deletion and anergy and have the ability to induce Treg cells. Both anti-CD28 and anti-CD154 treatments can significantly increase the number and function of MDSCs, suggesting that costimulatory blockade has a positive regulatory effect on MDSCs [25, 31]. Abatacept (CTLA-4Ig), the basis for the second-generation belatacept, was commonly used for treatment of patients with rheumatoid arthritis (RA) [132]. Previous studies showed that CTLA4-Ig downregulates the production of proinflammatory cytokines in synovial macrophages from RA patients or monocyte-derived macrophages from healthy donor cocultured with activated T cells [133, 134]. It also increases the absolute numbers of monocytes in RA patients after treatment. Monocytes from these patients showed reduced expression of adhesion molecules and displayed reduction in endothelial adhesion and transendothelial migration. Monocytes from healthy donors pretreated with CTLA-4Ig showed similar results [135]. We have no knowledge about belatacept or CTLA-4Ig on MDSCs so far, which requires to be studied.

## 5. Conclusion

MDSCs can be induced in different transplant animal models and clinical transplant cases. More importantly, the prolonged graft survival or transplant tolerance by immune modulation in some cases is all or partially dependent on MDSCs. This suggests that MDSCs play an important role in the maintenance of immune suppression and tolerance in certain situations, and targeting MDSCs may promote transplant tolerance induction. Some immunosuppressive agents enhance the function of MDSCs in transplantation significantly, but some will impair MDSC number and function. Considering the critical roles of MDSCs in transplant immune tolerance, we should put caution to the negative effects of certain immunosuppressive drugs on MDSCs, which may potentially block the tolerance induction in transplanted recipients. Understanding the impacts of immunosuppressive drugs on MDSCs may provide scientific guidance on the clinical optimal application of immunosuppressive agents.

## Figures and Tables

**Table 1:** MDSCs in transplantation.

Species	Years	Graft type	MDSC location	Functional subsets	In vitro assay	Immune modulation	Mechanism of action	Ref.
Human	2018	Intestine	PBMC, intestinal mucosa	PMN-MDSC, M-MDSC, e-MDSC	Anti-CD3/28/MLR	Tac + P/HC + (MMF)	—	[28]
Human	2014	Kidney	PBMC	Total	No	Tac(CsA) + P + (MMF)	—	[27]
Human	2013	Kidney	PBMC	M-MDSC, e-MDSC	MLR	Tac + P + MMF	—	[46]
Human	2015	Allo-HSCT	WBC	M-MDSC, PMN-MDSC	Anti-CD3/28	Tac + methotrexate	iNOS/Arg1	[48]
Human	2015	Haplo-identical allo-HSCT	WBC	M-MDSC, PMN-MDSC, e-MDSC	—	CsA + MTX + MMF	—	[49]
Human	2013	Allo-HSCT	PBMC	M-MDSC	Anti-CD2/CD3/CD28-stimulating bead	Tac(CsA) + MTX	IDO	[46]
Mouse	2003	BMT	Spleen	Total	MLR	Irradiation	iNOS	[50]
Mouse	2004	BMT	Spleen	Total	MLR	Irradiation SHIP KO	—	[54]
Mouse	2011 2012	Parental splenocyte to F1	Spleen	M-MDSC, PMN-MDSC	OT1 splenocytes with OVA/MLR	—	iNOS	[59] [60]
Mouse	2018	Heart	Spleen	M-MDSC	Anti-CD3/28	Dex	iNOS	[90]
Mouse	2015	Heart	Graft	CD11b^+^CD115^+^Ly6C^lo^, Ly6G^−^CD169^+^	MLR	Anti-CD40L	IL-10	[42]
Mouse	2010	Heart	Graft	M-MDSC	Anti-CD3/28	Anti-CD40L + DST	iNOS	[25]
Mouse	2015	Heart	Spleen	M-MDSC, PMN-MDSC	Anti-CD3/28	Rapa	iNOS	[30]
Mouse	2016	Skin	Spleen	M-MDSC (inhibition by rapa)	Anti-CD3/28	Rapa	iNOS	[29]
Mouse	2016	Skin	Spleen	Total	Anti-CD3/28	CsA	iNOS	[103]
Mouse	2014	Skin	Spleen	Total	MLR	Dex	iNOS	[89]
Mouse	2014	Skin	Spleen	Total	MLR	CsA	IDO	[102]
Mouse	2012	Heart	Graft	CD11b^+^IDO^+^	—	ECDI-fixed donor splenocyte + (rapa)	IDO	[44]
Rat	2008	Kidney	Blood	CD6^−^NKRP-1^+^CD80/86^+^	MLR	Anti-CD28	iNOS	[31]
Mouse	2011	Heart	Graft	M-MDSC	No	IL-33	—	[36]
Mouse	2012	Skin	Spleen	Total	MLR	Smad3-KO	iNOS	[34]
Mouse	2008	Skin	Spleen	Total	Anti-CD3/28	ILT2-TG	Arg1	[33]

TAC: tacrolimus; CsA: cyclosporin A; P: prednisolone/prednisone; HC: hydrocortisone; MMF: mycophenolate mofetil; MTX: methotrexate; MP: methylprednisolone.

**Table 2:** Immunosuppressive drugs on MDSCs.

Class	Drugs	Years	Disease	MDSC subsets/expansion/function	Mechanism	In vitro induction	Ref.
CSs	Dex	2017	Immunological hepatic injury	Total/?/suppress T cell proliferation in MLR↑, production of TNF*α*↓, IL-10↑	GR↑ → HIF1↓ → glycolysis↓ → NO↑	—	[88]
	Dex	2014	Skin Tx	Total/spleen↑, graft↑/suppress T cell proliferation in MLR↑	GR → iNOS↑ → Th1 to Th2	—	[89]
	MP	2018	Intestinal Tx	M, PMN, e-MDSC/PMBC↑/suppress T to donor intestinal epithelial organoids	—	Gm-CSF + IL-6 + G-CSF + (MP)	[28]
	Dex	2018	Heart Tx	M-MDSC/BM induction↑/suppress T proliferation by ConA, CXCR2↑	GR↑ → iNOS↑	Gm-CSF + (Dex)	[90]

mTORi	Rapa	2014	Immunological hepatic injury	M-MDSC/liver↑/suppress T cell proliferation in MLR↑, CXCR2↑	iNOS↑ → recruitment to the liver↑	—	[111]
	Rapa	2014	Tumor in SIRT1 KO mice	Total/?/suppress T cell proliferation in MLR↑, production of NO↓, TNF*α*↓, Arg1↑, IL-10↑	Glycolysis↓	—	[113]
	Rapa	2015	Heart Tx	M, PMN-MDSC/spleen↑/suppress T cell proliferation by anti-CD3/28↑	iNOS↑, Arg1↑, Treg↑	—	[30]
	Rapa	2016	Skin Tx, tumor	M-MDSC/BM induction↓, spleen↓/suppress T cell proliferation by anti-CD3/28↓	Glycolysis↓, iNOS↓	Gm-CSF + (rapa)	[29]
	Rapa	2016	Immunological hepatic injury	Total/liver↑/suppress T cell proliferation in MLR↑, CXCR2↑	HIF1*α*↓, glycolysis↓, iNOS↑	Gm-CSF + (rapa)	[112]
	Rapa	2017	Immunological kidney injury	M, PMN-MDSC/spleen↑, kidney↑/suppress T cell proliferation by anti-CD3/28↑	Runx1↓ → iNOS↑, Arg1↑	Gm-CSF + IL-6 + (rapa)	[114]

CNIs	CsA	2014	Skin Tx	Total/spleen↑/suppress T cell proliferation in MLR↑, CXCR2↑, TNF*α*↓, IL-10↑, Th1 to Th2↑	NFAT↓ → IDO↑	—	[102]
	CsA	2016	Skin Tx	Total/spleen↑, BM induction↑/suppress T cell proliferation in MLR↑	iNOS↑	Gm-CSF + (CsA)	[103]

Costimulatory molecules	Anti-CD28	2008	Kidney Tx	Total/blood↑/suppress T cell proliferation in MLR↑	iNOS↑	—	[31]
	Anti-CD40L	2010	Heart Tx	M-MDSC/graft↑/suppress T cell proliferation by anti-CD3/28↑	IFN-*γ*R → STAT1 → iNOS↑	—	[25]
	Anti-CD40L	2015	Heart Tx	MDSC-derived M*φ*/graft ↑/suppress T cell proliferation in MLR↑	DC-SIGN, TLR-4 → IL-10↑	—	[42]
